# Impacts of the Seattle Sweetened Beverage Tax on the Perceived Healthfulness of Sweetened Beverages

**DOI:** 10.3390/nu14050993

**Published:** 2022-02-26

**Authors:** Lauren Sawyer, Vanessa M. Oddo, Amanda Fretts, Melissa A. Knox, Nadine Chan, Brian E. Saelens, Jessica C. Jones-Smith

**Affiliations:** 1 Nutritional Sciences Program, School of Public Health, University of Washington, Seattle, WA 98195, USA; 2 Department of Kinesiology and Nutrition, University of Illinois Chicago, Chicago, IL 60612, USA; voddo@uic.edu; 3 Department of Epidemiology, School of Public Health, University of Washington, Seattle, WA 98195, USA; amfretts@uw.edu (A.F.); nadine.chan@kingcounty.gov (N.C.); jjoness@uw.edu (J.C.J.-S.); 4 Department of Economics, University of Washington, Seattle, WA 98195, USA; knoxm@uw.edu; 5 Public Health—Seattle & King County, Assessment, Policy Development and Evaluation Division, Seattle, WA 98104, USA; 6 Seattle Children’s Research Institute, Seattle, WA 98101, USA; brian.saelens@seattlechildrens.org; 7 Department of Pediatrics, School of Medicine, University of Washington, Seattle, WA 98105, USA; 8 Department of Health Systems and Population Health, School of Public Health, University of Washington, Seattle, WA 98195, USA

**Keywords:** sweetened beverages, beverage tax, health perceptions, food policy, health policy

## Abstract

Sweetened beverage taxes are associated with significant reductions in the purchase of sweetened beverages. However, it is unclear whether these taxes play a role in shifting perceptions about sweetened beverages and their health impacts. We utilized pre- and post-tax survey data collected from residents in Seattle, WA, a city that implemented a sweetened beverage tax in 2018 and from residents in an untaxed comparison area. We used income-stratified difference-in-difference linear probability models to compare net changes in the perceived healthfulness of overall sweetened beverage consumption and of different types of sugary beverages over time and across income groups. We found significant increases in the percentage of Seattle respondents with lower incomes who agreed that sweetened beverage consumption raises the risk of diabetes (DD = 9 percentage points (pp) (95% CI: 5 pp, 13 pp); *p* = 0.002), heart disease (DD = 7 pp (95% CI: 2 pp, 12 pp); *p* = 0.017), and serious health problems (DD = 12 pp (95% CI: 5 pp, 19 pp); *p* = 0.009), above and beyond changes in the comparison area. The most prominent changes in perceived health impacts of sweetened beverages were found among lower-income Seattle respondents, while fewer changes were found among higher-income Seattle respondents. Future work could examine the relationship between exposure to pro-tax messaging and changes in consumer perceptions of sweetened beverages.

## 1. Introduction

Over the past decade, cities and countries around the world have implemented sweetened beverage taxes with the goal of raising revenue and decreasing sweetened beverage consumption, which has been linked to diabetes and heart disease [1]. Regions that have implemented a sweetened beverage tax have seen overall declines in the total volume of sweetened beverage purchases [2], with some studies (primarily of Mexico’s tax) finding greater reductions in purchases among households with lower socioeconomic status [3,4,5]. These reductions may be related to increases in sweetened beverage prices, resulting from the tax mostly being passed through to consumers [6]. Indeed, the increase in price is hypothesized to be the primary way in which sweetened beverage taxes work to decrease purchasing, and consumers with lower incomes may be especially sensitive to these price changes and respond accordingly. However, the media coverage associated with these taxes, along with any explicit pro- or anti-tax media campaigns, may also serve to reduce sweetened beverage consumption by increasing awareness of its harms, and thus open a second pathway (i.e., an “awareness” pathway) by which the existence of sweetened beverage taxes may operate to change public preferences for sweetened beverages [7].

Evidence against this “awareness pathway” developing in cities with sweetened beverage taxes was documented in the evaluation of Chicago’s short-lived beverage tax, whereby the purchasing of taxed beverages sharply declined immediately after implementation of the tax but returned to pre-tax levels shortly after the tax was repealed [8]. However, there is some suggestive evidence consistent with increased awareness from the evaluation of Seattle’s Sweetened Beverage Tax. We found that upon tax implementation, sweetened beverage consumption decreased substantially among lower-income families in Seattle, as well as low-income families living in nearby cities that share the same media market as Seattle [9]. Additionally, Cawley et al. [10] noted that studies of beverage taxes that use a nearby comparison area find smaller differential changes in beverage consumption between taxed and nearby untaxed areas compared to studies that use a more distant comparison area; this could be due to multiple causes, including sharing a media market, confusion about which areas are taxed, and being exposed to messaging about the tax.

To better understand whether increased awareness may play a role in changing behavior in response to beverage taxes, we conducted a survey to evaluate the impact of Seattle’s Sweetened Beverage Tax on beliefs, attitudes, and norms. We used pre- and post-tax data collected in Seattle and non-taxed comparison areas outside of the Seattle media market to determine whether the beverage tax influenced perceptions of health risks associated with overall consumption of sweetened beverages, and if this differed by income. Additionally, we aimed to assess whether the beverage tax had an impact on changing consumer perceptions of the healthfulness of different types of sweetened beverages (i.e., sports drinks, juice drinks, etc.) since people may become newly aware that a variety of drink types have enough sugar to be subject to the tax [11,12].

In Seattle, we previously found income-differentiated support for the tax and the perception that the tax would benefit public health [13], providing early indications that Seattle consumers may perceive the healthfulness of sweetened beverages differently, depending on their income. Additionally, we explore these associations by income since lower income populations may be more responsive to price changes induced by the sweetened beverage tax. As our current understanding of tax impacts mostly centers around tax pass-through and price responsiveness, insight into consumer awareness and health perceptions will be important for public health policies and research going forward, as these factors may alter the effects of a price change.

## 2. Materials and Methods

### 2.1. Data Collection

In January 2018, the City of Seattle implemented a 1.75 cent per ounce tax on the distribution of sweetened beverages. We administered a pre-tax survey, from October to December 2017, via telephone and online to residents of Seattle, WA, and non-taxed comparison areas, which included Arlington, VA, Bethesda and Rockville, MD (combined), and Minneapolis, MN. A follow-up survey was administered to a new survey population by telephone and online, approximately two years following tax implementation between September and November 2019. At each time point, we aimed to recruit 800 participants each from Seattle and the comparison area. The survey design, recruitment process, and selection of comparison cities have been described previously [13]. Briefly, we chose the comparison areas based on places in the US with similar demographic characteristics and political leanings to Seattle, and where there was no existing new or local sweetened beverage tax. We administered a repeated cross-sectional survey, using quotas to ensure racial/ethnic representation approximately equivalent to each city’s distribution and oversampling people with lower incomes to enable income-specific comparisons. Survey questions pertained to demographics, opinions of the tax, and the perceived economic and health impacts of tax implementation and sweetened beverage consumption.

### 2.2. Primary Variables

#### 2.2.1. Independent Variables

The primary independent variable was exposure to the beverage tax, wherein the “treatment” group consisted of Seattle residents and the “control” group consisted of residents in the comparison area. Additionally, we included time as a second independent variable to examine changes in the perceived health impacts of sweetened beverages between pre- and post-tax periods.

#### 2.2.2. Dependent Variables

The dependent variables of interest included a summary score of respondents’ responses to 11 questions about perceived health impacts of sugary beverages and added sugar, and perceived healthfulness of different sugary beverage types. In addition, we examined each of these 11 outcomes separately. Specifically, there were 6 questions about the perceived health impacts of sweetened beverage consumption (e.g., serious health problems, dental health problems, obesity, diabetes, and heart disease) and the perceived healthfulness of added sugar (not specific to beverages), and 5 questions about the healthfulness of different types of sweetened beverages (i.e., soda, fruit drinks, sports drinks, sweetened tea and coffee, and energy drinks).

Survey respondents were asked to indicate on a scale how much they agreed (strongly disagree, somewhat disagree, somewhat agree, strongly agree, or don’t know) with the statements: “*Drinking sugary drinks causes serious health problems*”, and *“Drinking sugary drinks significantly raises a person’s chances of [dental health problems, obesity, diabetes, heart disease]*”. Respondents were also asked to indicate on a scale whether they agreed that excessive sugar from sources not limited to sugary beverages can lead to serious health problems. Responses to these questions were then dichotomized into a two-category variable: ‘strongly agree’ and ‘somewhat agree’ were combined into a new ‘agree’ category, while ‘strongly disagree’ and ‘somewhat disagree’ were combined into a new ‘disagree’ category.

Respondents were also asked to indicate whether they thought “Regularly drinking (soda, fruit-flavored drinks, sports drinks, sweetened teas or coffees, energy drinks) affects a person’s chances of developing health problems like diabetes or becoming overweight”. Answer options included ‘Doesn’t increase’, ‘Probably increases’, ‘Definitely increases’, or ‘Don’t know’. Responses to these questions were also dichotomized into a two-category variable: ‘doesn’t increase’ remained its own category, while ‘probably increases’ and ‘definitely increases’ were combined into a new ‘increases’ category.

We limited our analysis to only those who answered all the questions about health impacts, added sugar, and drink type to ensure a consistent sample between each model. People who answered ‘Don’t know’ to any of the aforementioned questions were also excluded from the primary analysis.

To measure respondents’ overall health perceptions of sweetened beverages in response to the tax, we created a summed score using the 11 questions that asked about added sugar, negative health outcomes (serious health problems, dental health problems, diabetes, obesity, and heart disease), and drink type (fruit drinks, soda, sports drinks, sweetened tea/coffee, energy drinks). Respondents received a −1 if they disagreed that sweetened beverages negatively impact health, and a 1 if they agreed that sweetened beverages negatively impact health. Scores ranged from −11 to 11, where higher scores represent the perception that sweetened beverages negatively impact health.

#### 2.2.3. Covariates

We stratified our analyses by income based on prior evidence that post-tax changes in sweetened beverage purchasing may differ by income and our a priori interest in income differences (resulting in our oversampling of lower-income households) [3,4,5]. Respondents were asked to report their household size and annual household income, which was then used to create a dichotomous income variable: “lower income” was categorized as having an income <260% of the federal poverty line (FPL), and “higher income” was categorized as having an income ≥260% FPL, in alignment with subsidized health care tiers (Apple Care) in Washington state. We controlled factors that we hypothesized to be associated with the exposure (living in Seattle) and the outcome (health perceptions), including race/ethnicity (Non-Hispanic White, Non-Hispanic Black/African American, Non-Hispanic Asian, Non-Hispanic Other, Hispanic), education (some high school, completed high school, some college or vocational training, completed college or university, completed graduate or professional degree), age (18–30, 31–40, 41–50, 51–64, 65+), sex (male or female), survey mode (phone or web), and political affiliation (Democrat, Independent, Republican, other, don’t know).

### 2.3. Statistical Analysis

We created two different weights to use in this analysis. First, we used the raking method to create population weights, so that our study samples were representative of their respective city’s demographics (race/ethnicity, sex, age, and annual median household income), according to the 2017 5-year American Community Survey [13,14]. We then stratified by income (<260% FPL and ≥260% FPL) and created propensity score weights for each stratum to minimize group differences across time and location (with exception to Table 1, which used non-income-stratified propensity scores since these are presented for the overall sample rather than income-stratified); other than income, all covariates plus information on marital status were used in the creation of propensity score weights. This method weighted each group (Seattle pre-tax, comparison pre-tax, Seattle post-tax, comparison post-tax) in such a way as to allow resemblance and comparison to the Seattle pre-tax group [15]. The population weights and propensity score weights were then multiplied together to create a combined weight for analysis.

To examine the changes in the perceived health impacts of our summary score and each individual outcome, we separately modeled 12 different outcomes for low- and high-income individuals, resulting in 24 difference-in-difference linear probability regression models. Difference estimates across timepoints represent the change from pre-tax to post-tax in either Seattle or the comparison areas in the percentage of the population who agreed with the health perception statements. Difference-in-difference estimates represent changes in the percentage of the Seattle population over time after accounting for the changes seen in the comparison area over the same period of time. The coefficients produced by these models were then multiplied by 100 and interpreted as percentage point changes, with the exception of the summary scores. All models use this general format:

Y_it_ = β_0_ + β_1_(city)_i_ + β_2_(time)_t_ + β_3_(city ∗ time)_it_ + γX_it_ + e_it_ (1)

where Y_it_ is the outcome of interest for person i at time t, and B_3_ is the difference-in-difference estimator and X is a vector of covariates. In our primary analyses, we set α to 0.05. However, we also corrected for multiple comparisons using a Bonferroni correction based on 11 outcomes of interest (not counting the summary score) among both lower and higher income participants (0.05/22 = 0.002); as the outcomes are correlated, this correction is likely conservative. In sensitivity analyses, we examined whether there were changes in the percentage of the population who answered ‘Don’t know’ to each of the questions above using multinomial logit models.

Statistical analysis was conducted using Stata 15.1 (StataCorp LP, College Station, TX, USA).

## 3. Results

### 3.1. Demographic Characteristics

**Figure 1:**
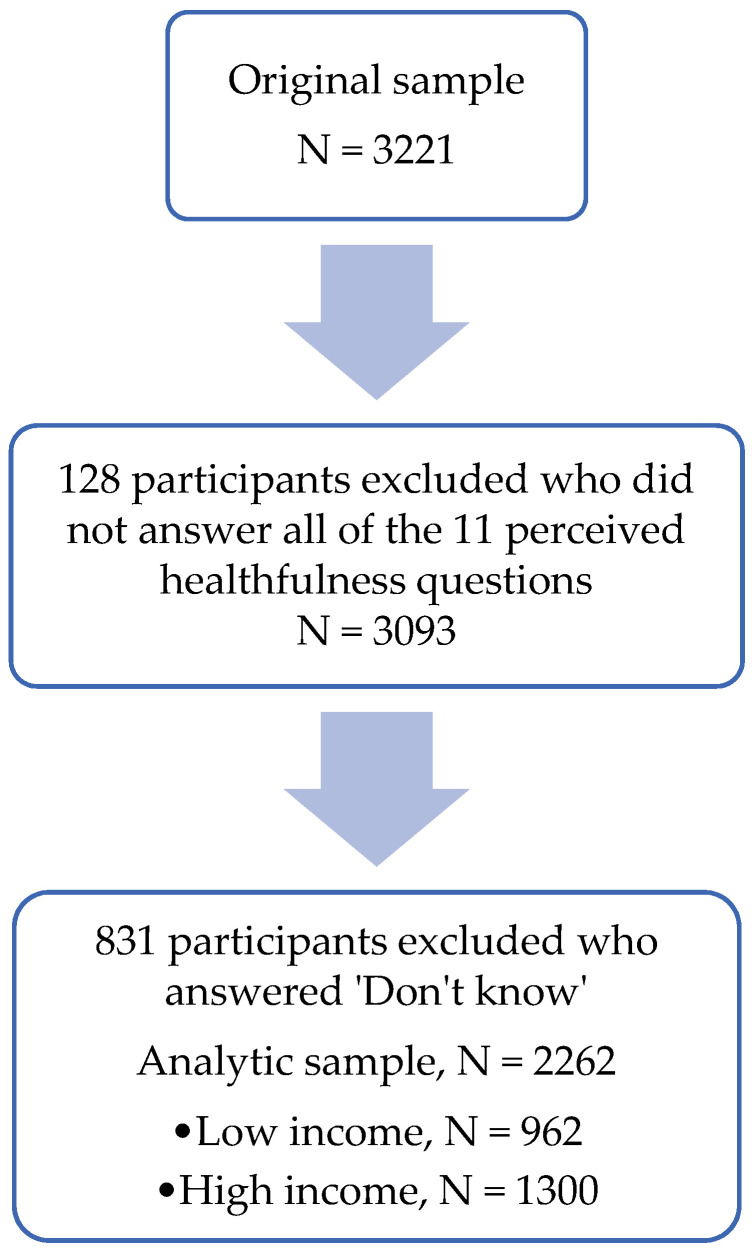
Study Sample Flowchart.

Our sample consisted of 3221 respondents. After limiting the sample to include only those who answered all 11 perceived healthfulness questions and excluding those who answered ‘Don’t know’, our final analytic sample consisted of 2262 respondents (Figure 1). Our weighted sample showed a somewhat consistent distribution of demographic characteristics between pre-tax and post-tax time points and between Seattle and comparison areas (Table 1). The majority of respondents in our sample identified as Non-Hispanic White, Democrat, and higher income. The distribution of age and sex were similar across tax exposure and time.

### 3.2. Summary Score of Health Perceptions among Low- and High-Income Respondents in Seattle and Comparison Areas

Among lower-income individuals, the difference-in-difference (DD) estimate indicated that the tax was associated with significant increases in the perceptions that sweetened beverages negatively impact health among Seattle versus comparison area respondents (DD = 1.05 (95% CI: 0.57, 1.54); *p* = 0.004) (Table 2). Among higher-income individuals, the difference-in-difference estimates suggest that there was no significant change in the perception that sweetened beverages negatively impact health (DD = −0.00 (95% CI: −0.61, 0.60); *p* = 0.985).

### 3.3. Differences in Perceived Health Impacts of Sweetened Beverage Consumption among Low-Income Respondents in Seattle and Comparison Areas

The difference-in-difference estimates showed that the percentage of Seattle respondents with lower incomes who agreed that sweetened beverage consumption increases the risk of various negative health outcomes differed significantly from respondents in comparison areas (Table 3). The percentages at pre-tax levels in addition to the changes over time are represented in Supplementary Tables S1 and S2. Broadly, the pre-tax percentages of Seattle and comparison area respondents with lower incomes who linked sweetened beverages with negative health impacts ranged from 79–89 percent and 84–94 percent, respectively (Table S1); these ranges are narrower and trend lower than those of higher income respondents (Table S2). Significant relative increases were found in the percentage of Seattle respondents (versus comparison area respondents) who agreed that sweetened beverage consumption increases the risk of serious health problems (DD = 12 percentage points (pp) (95% CI: 5 pp, 19 pp); *p* = 0.009), diabetes (DD = 9 pp (95% CI: 5 pp, 13 pp); *p* = 0.002), and heart disease (DD = 7 pp (95% CI: 2 pp, 12 pp); *p* = 0.017). Additionally, the percentage of Seattle respondents who agreed that added sugar affected the risk of developing health problems increased significantly compared to the change in the comparison area (DD = 20 pp (95% CI: 15 pp, 24 pp); *p* < 0.001). Findings for diabetes and added sugar remained significant after accounting for Bonferroni corrections.

Estimates from our sensitivity analysis show that, for most of the health impact questions, the change in Seattle respondents with lower incomes who answered ‘Don’t know’ did not differ significantly from those in the comparison area. The one exception was regarding dental health (DD = 2.59 (95% CI: 0.30, 4.88); *p* = 0.035), indicating an increase in the probability of reporting ‘Don’t know’ to the question that asked whether respondents agreed or disagreed that drinking sugary drinks increases the likelihood of dental health problems, compared to the probability of reporting that they agreed. This estimate did not remain significant after Bonferroni adjustment (Table S3).

### 3.4. Differences in Perceived Health Impacts of Sweetened Beverage Consumption among High-Income Respondents in Seattle and Comparison Areas

Pre-tax percentages of Seattle and comparison area respondents with higher incomes, who agreed that sweetened beverage consumption increases the likelihood of negative health outcomes, ranged from 79–95 percent and from 79–96 percent, respectively (Table S2). Significant changes were found for the impact of the tax on the percentage of higher income respondents in Seattle versus the comparison area on whether sweetened beverage consumption increases the risk of serious health problems (DD = −7 pp (95% CI: −11 pp, −3 pp); *p* = 0.009) and heart disease (DD = −7 pp (95% CI: −13 pp, −1 pp); *p* = 0.038). However, these findings were not significant after applying a Bonferroni correction. Sensitivity analysis indicated that the change in the proportion of Seattle respondents with higher incomes who answered ‘Don’t know’ to the above health impact questions did not differ from the changes among those in the comparison area (Table S3).

### 3.5. Differences in Perceived Healthfulness of Sweetened Beverage Types among Low-Income Respondents in Seattle and Comparison Areas

The difference-in-difference estimates suggest that the increase in the percentage of Seattle respondents with lower incomes who perceived fruit drinks, soda, and sports drinks as having negative health impacts was not significantly different from the changes among comparison area respondents with lower incomes; a non-statistically significant decrease was found for energy drinks (Table 4). However, the percentage of Seattle respondents who agreed that sweetened tea/coffee increases the risk of developing health problems increased less compared to changes in the percentage of comparison area respondents (DD = −7 pp (95% CI: −13 pp, −1 pp); *p* = 0.034). While this finding was significant at 0.05, it was not significant after applying a Bonferroni correction. The pre-tax percentages of respondents in Seattle and the comparison area ranged from 79–91 percent and 79–93 percent, respectively (Table S1); these ranges are broader and trend lower than those of higher income respondents (Table S2). In our sensitivity analysis, for most drink type questions, the changes in the proportion of Seattle respondents with lower incomes who answered ‘Don’t know’ did not differ significantly from changes in the comparison area. The one exception was with regard to sports drinks (DD = 0.42 (95% CI: 0.00, 0.83); *p* = 0.049) (Table S4), indicating an increase in the probability of reporting ‘Don’t know’ to the question that asked respondents whether consuming sports drinks increases or doesn’t increase the likelihood of health problems, compared to the probability of reporting that they increase the likelihood.

### 3.6. Differences in Perceived Healthfulness of Sweetened Beverage Types among High-Income Respondents in Seattle and Comparison Areas

Comparing the change over time in the perceptions for Seattle respondents with higher incomes to the change over time in the same perceptions among comparison area respondents with higher incomes, the difference-in-difference estimates were not statistically significant (Table 4), indicating that the tax did not differentially impact higher income populations exposed versus unexposed to the tax. The pre-tax percentages of respondents in Seattle and the comparison area ranged from 85–95 percent and 83–96 percent, respectively (Table S2).

Sensitivity analyses indicate that the changes in the proportion of Seattle respondents with higher incomes who answered ‘Don’t know’ to the beverage type questions differed significantly from changes among comparison area respondents regarding sports drinks (DD = −0.37 (95% CI: −0.73, −0.00); *p* = 0.050), sweetened tea/coffee (DD = −0.61 (95% CI: −0.82, −0.40); *p* = 0.001), and energy drinks (DD = −0.30 (95% CI: −0.53, −0.06); *p* = 0.024) (Table S4). These estimates indicate decreases in the probability of responding ‘Don’t know’ to questions that asked whether consuming these beverages increases or doesn’t increase the likelihood of health problems, compared to the probability of reporting that they increase the likelihood.

## 4. Discussion

The current study provides further insight into income-related differences in perceptions about sweetened beverage healthfulness and how these perceptions might change in response to a beverage tax. We assessed changes in the perceptions of the healthfulness of sweetened beverage consumption and sweetened beverage types in Seattle and comparison areas across higher and lower income groups, before and after a sweetened beverage tax was implemented in Seattle. Overall, our findings suggest that the institution of the tax was associated with increases in the perception that sweetened beverages negatively impact health among lower income respondents. Regarding perceptions of individual health outcomes, we found significant increases in the percentage of Seattle respondents with lower incomes who agreed that sweetened beverage consumption increased the likelihood of health problems, as compared to respondents with higher incomes and after accounting for analogous changes in the comparison groups. Conversely, across both income groups, the changes in the percentage of Seattle respondents who agreed that different sweetened beverage types increase someone’s risk of developing health problems did not differ significantly from the changes in the comparison area, with the exception of sweetened tea/coffee.

The results from prior studies have suggested possible income-based divergences in purchasing and consumption responses to the tax [3,4,5,16], and it is unclear whether this may be related to the tax signaling (i.e., implying to consumers) the negative health impacts related to sweetened beverages, thus raising consumer awareness. One study in Berkeley found that 68% of all respondents reported having an awareness of the tax and that groups with lower incomes reduced their sweetened beverage consumption by 21%, which coincided with robust media campaigns around the tax [16]. While this study did not assess the correlation between tax awareness and consumption, results from a study of Mexico’s sweetened beverage tax and possible signaling effects by Alvarez-Sanchez and colleagues [17] showed that those who reported knowing a beverage tax had been implemented were 30% more likely to self-report decreases in consumption [17]. This contrasts with the findings by Powell et al. [8], which showed that, in Chicago, the purchasing of sweetened beverages rebounded to the pre-tax levels shortly after the repeal of the tax; this suggests that the price effects of the tax impact consumer behavior more so than any lasting education effect of the tax or tax media. Notably, this rebound in purchasing could have been related to the fact that the beverage tax was only in place for 4 months prior to its repeal [8]. If tax presence is impacting consumer perceptions of health through signaling rather than price impacts, it is possible that 4 months would not be enough to evoke significant changes in perception. Additionally, Chicago’s pro-tax campaign focused mostly on revenue generated from the tax, rather than public health-focused messaging [8], which could represent a lost opportunity for health education during the tax campaign. Furthermore, the tax in Cook County, IL (Chicago), taxed both sugar- and artificially-sweetened beverages, and consumers may not have perceived the tax as one relating to health, or health outcomes associated with sugar consumption.

Our findings that the tax was associated with increases in the perception that sweetened beverages negatively impact health among lower-income populations in Seattle might be explained by the local context and differently targeted pro-tax campaigns. While it is possible that our findings are consistent with the tax signaling the unhealthfulness of sugary beverages and this being more salient for lower versus higher income residents, it might also be related to the increased pro-tax public health messaging aimed at communicating the ill-health effects of sweetened beverages among groups with lower incomes. While there was not a city-wide effort to promote the beverage tax in Seattle, grass-roots community-based organizations, such as the local organization Got Green, specifically conducted outreach to lower-income audiences and communities of color for pro-tax messaging that communicated the negative health impacts of sweetened beverages [18]. Thus, it is possible that the combined exposure to these messages along with the presence of the tax may have influenced the perceived health impacts of sweetened beverages among lower-income groups, implying the importance of public health-based tax messaging. For other cities looking to implement taxes, these outreach campaigns may be a useful model to follow for providing information about a tax and its intended benefits, especially among lower income populations.

Our study is limited by the repeated cross-sectional design, which prevented us from following the same participants over time. However, our use of population and propensity score weights, and the ability to adjust for factors that may residually differ across Seattle and the comparison area resulted in estimates that are “doubly robust” [19] and are more likely to account for income and location differences between groups over time. The generalizability of our results may be somewhat limited by the location of our study in Seattle, despite the inclusion of a comparison group. However, some findings, such as increased awareness of the ill-health effects of sugary beverages among lower income populations, could conceivably be found in other places considering beverage taxes or that have existing taxes and are worth further research. Additionally, we conducted the survey in three languages (English, Spanish, Vietnamese); however, we were only able to offer the Vietnamese version online, whereas the English and Spanish versions were offered online and by telephone. Lastly, our summary score was created by scoring each outcome and combining them together, but we did not test the validity of this measure to act as a summary of the component parts.

## 5. Conclusions

Overall, we found that changes in the perceived health impacts of sweetened beverage consumption and various sweetened beverage types increased significantly in groups with lower incomes in Seattle, above and beyond the comparison area, or in the groups with higher incomes. Future work could examine the relationship between health perceptions and changes in consumption and/or purchasing volumes in Seattle, as well as correlations between exposure to pro-tax messaging and changes in consumer perceptions of sweetened beverages.

## Figures and Tables

**Table 1:** Demographic Characteristics of Samples ^a,b^.

	Seattle		Comparison	
	Pre-Tax (*n* = 610)	Post-Tax (*n* = 573)	Pre-Tax (*n* = 536)	Post-Tax (*n* = 543)
	*n* (%)	*n* (%)	*n* (%)	*n* (%)
Age				
18–30	103 (21.0%)	110 (21.0%)	110 (20.5%)	145 (23.1%)
31–40	114 (21.8%)	120 (24.3%)	132 (25.4%)	118 (23.6%)
41–50	109 (21.6%)	97 (20.3%)	79 (19.6%)	66 (15.6%)
51–64	125 (24.0%)	130 (20.6%)	101 (23.4%)	97 (22.8%)
65+	159 (11.6%)	116 (13.9%)	114 (11.1%)	117 (14.9%)
Sex				
Male	243 (50.3%)	248 (52.2%)	295 (49.8%)	169 (49.0%)
Female	367 (49.7%)	325 (47.8%)	241 (50.2%)	374 (51.0%)
Race/Ethnicity				
Non-Hispanic White	430 (66.0%)	401 (66.7%)	335 (63.3%)	346 (66.7%)
Non-Hispanic Black/African American	36 (5.3%)	42 (8.5%)	40 (6.7%)	69 (5.5%)
Non-Hispanic Asian	49 (14.2%)	57 (13.7%)	57 (14.7%)	53 (13.2%)
Non-Hispanic Other	54 (7.1%)	33 (5.1%)	21 (6.8%)	35 (8.8%)
Hispanic	41 (7.4%)	40 (6.0%)	83 (8.5%)	40 (5.8%)
Income				
Lower income (<260% FPL)	269 (36.4%)	228 (32.5%)	233 (44.4%)	232 (33.6%)
Higher income (≥260% FPL)	341 (63.6%)	345 (67.5%)	303 (55.6%)	311 (66.4%)
Education				
Some high school	16 (4.0%)	9 (4.3%)	29 (5.7%)	8 (4.5%)
Completed high school	55 (9.8%)	62 (9.4%)	61 (9.8%)	71 (9.9%)
Some college or vocational training	142 (22.0%)	164 (21.3%)	124 (22.7%)	135 (22.0%)
Completed college	223 (37.4%)	193 (36.9%)	166 (36.9%)	218 (39.0%)
Completed graduate degree	174 (26.8%)	145 (28.1%)	156 (24.9%)	111 (24.7%)
Political Affiliation				
Democrat	353 (55.4%)	323 (54.8%)	240 (56.4%)	275 (51.5%)
Independent	174 (30.3%)	152 (28.1%)	160 (27.3%)	127 (29.0%)
Republican	47 (8.4%)	56 (10.1%)	86 (8.0%)	74 (9.9%)
Other	9 (1.8%)	18 (1.7%)	13 (2.5%)	11 (2.7%)

^a^ The N’s are unweighted counts, while the percentages were weighted using a combined population weight (created using the raking method) and propensity score weight not based on income strata to improve representation of city demographics within each study sample. ^b^ These demographics represent the sample of respondents who answered all 11 of the perceived healthfulness questions.

**Table 2:** Differences in Summary Health Scores Among Seattle and Comparison Area Respondents with Lower and Higher Incomes ^a–d^.

	Lower Income (*n* = 962)			Higher Income (*n* = 1300)		
	Seattle Difference (95% CI)	Comparison Difference (95% CI)	DD (95% CI)	Seattle Difference (95% CI)	Comparison Difference (95% CI)	DD (95% CI)
Summary Health Score	**0.64**	**−0.42**	**1.05**	**0.19**	0.19	−0.00
	**(0.41, 0.86)**	**(−0.81, −0.03)**	**(0.57, 1.54)**	**(0.11, 0.26)**	(−0.43, 0.81)	(−0.61, 0.60)

^a^ CI = Confidence Interval; DD = Difference-in-difference, ^b^ Bolded values indicate significance at *p* < 0.05, ^c^ Lower income is defined as having an income <260% FPL; Higher income is defined as having an income ≥260% FPL. ^d^ The estimates in these models were created using population weights combined with propensity score weights, and represent changes in the average summary score. Race/ethnicity, education, age, sex, survey mode, and political affiliation were controlled for in both models.

**Table 3:** Income-Stratified Pre- to Post-tax Differences in the Percentage of Those Perceiving Negative Health Consequences of Sweetened Beverage Consumption in Seattle and Comparison Areas ^a–e^.

Health Impacts	Lower Income (*n* = 962)			Higher Income (*n* = 1300)		
	Seattle Difference (95% CI)	Comparison Difference (95% CI)	DD (95% CI)	Seattle Difference (95% CI)	Comparison Difference (95% CI)	DD (95% CI)
Drinking sugary drinks causes serious health problems	1	**−11**	**12**	−2 *	**5**	**−7**
	(−1, 2)	**(−18, −4)**	**(5, 19)**	**(−2, −1)**	**(1, 10)**	**(−11, −3)**
Drinking sugary drinks significantly raises a person’s chances of dental health problems, including cavities and tooth decay	1 *	−4	5	2 *	−0	2
	**(1, 2)**	(−10, 3)	(−1, 12)	**(2, 2)**	(−10, 9)	(−7, 12)
Drinking sugary drinks significantly raises a person’s chances of obesity	5 *	2	3	4 *	1	3
	**(3, 7)**	(−3, 7)	(−2, 9)	**(3, 4)**	(−8, 9)	(−5, 12)
Drinking sugary drinks significantly raises a person’s chances of diabetes	**1**	**−8**	9 *	5 *	2	3
	**(0, 3)**	**(−12, −4)**	**(5, 13)**	**(4, 6)**	(−5, 8)	(−3, 9)
Drinking sugary drinks significantly raises a person’s chances of heart disease	**3**	−4	**7**	**2**	**9**	**−7**
	**(0, 5)**	(−10, 1)	**(2, 12)**	**(1, 3)**	**(3, 15)**	**(−13, −1)**
Consuming excessive amounts of sugar from any source can lead to health problems	11 *	**−9**	20 *	−2 *	−3	1
	**(9, 12)**	**(−13, −5)**	**(15, 24)**	**(−2, −1)**	(−8, 2)	(−3, 6)

^a^ CI = Confidence Interval; DD = Difference-in-difference. ^b^ Bolded values indicate significance at *p* < 0.05; values with an (*) indicate significance of *p* = 0.002, according to the Bonferroni correction. ^c^ Lower income is defined as having an income <260% FPL; Higher income is defined as having an income ≥260% FPL. ^d^ The estimates in these models were created using population weights combined with propensity score weights. Difference estimates represent changes in the percentage of the population over time, while differences-in-differences estimates represent changes over time in Seattle compared to the changes over time in comparison areas. Units for all estimates are percentage points rounded to the nearest whole number. Race/ethnicity, education, age, sex, survey mode, and political affiliation were controlled for in each model. ^e^ Estimates exclude respondents that answered ‘Don’t know’ to any of the health impact questions (serious health problems, *n* = 113; dental health problems, *n* = 79; obesity, *n* = 85; diabetes, *n* = 117; heart disease, *n* = 318; added sugar, *n* = 148).

**Table 4:** Income-Stratified Differences in Perceived Healthfulness of Sweetened Beverage Types in Seattle and Comparison Areas ^a–e^.

Sweetened Beverage Types	Lower Income (*n* = 962)			Higher Income (*n* = 1300)		
	Seattle Difference (95% CI)	Comparison Difference (95% CI)	DD (95% CI)	Seattle Difference (95% CI)	Comparison Difference (95% CI)	DD (95% CI)
Drinking fruit-flavored drinks affects a person’s chances of developing health problems	3 *	2	2	**−1**	−4	3
	**(2, 5)**	(−5, 8)	(−5, 8)	**(−2, −0)**	(−13, 4)	(−5, 12)
Drinking soda affects a person’s chances of developing health problems	**2**	−0	2	2 *	−2	4
	**(1, 4)**	(−6, 6)	(−4, 9)	**(2, 3)**	(−8, 4)	(−2, 9)
Drinking sports drinks affects a person’s chances of developing health problems	3 *	1	2	1	2	−1
	**(2, 3)**	(−7, 8)	(−6, 10)	(−1, 2)	(−1, 5)	(−5, 2)
Drinking sweetened teas or coffees affects a person’s chances of developing health problems	3 *	**10**	**−7**	**−1**	1	−1
	**2, 4)**	**(4, 17)**	**(−13, −1)**	**(−1, −0)**	(−2, 3)	(−3, 1)
Drinking energy drinks affects a person’s chances of developing health problems	**−1**	1	−2	−1	0	−1
	**(−3, −0)**	(−8, 10)	(−10, 6)	**(−1, −0)**	(−1, 2)	(−2, 0)

^a^ CI = Confidence Interval; DD = Difference-in-difference. ^b^ Bolded values indicate significance at *p* < 0.05; values with an (*) indicate significance of *p* = 0.002, according to the Bonferroni correction. ^c^ Lower income is defined as having an income <260% FPL; Higher income is defined as having an income ≥260% FPL. ^d^ The estimates in these models were created using population weights combined with propensity score weights. Difference estimates represent changes in the percentage of the population over time, while differences-in-differences estimates represent changes over time in Seattle compared to the changes over time in comparison areas. Units for all estimates are percentage points rounded to the nearest whole number. Race/ethnicity, education, age, sex, survey mode, and political affiliation were controlled for in each model. ^e^ Estimates exclude respondents that answered ‘Don’t know’ to any of the sweetened beverage type questions (fruit drinks, *n* = 227; soda, *n* = 149; sports drinks, *n* = 409; sweetened tea/coffee, *n* = 264; energy drinks, *n* = 364).

## Data Availability

The de-identified data presented in this study are available on request from the last author (J.C.J.-S.).

## Supplementary Materials

The following supporting information can be downloaded at: https://www.mdpi.com/article/10.3390/nu14050993/s1, Table S1: Pre- to Post-tax Levels and Differences in the Percentage of Lower-income Respondents Perceiving Negative Health Consequences of Sweetened Beverage Consumption and Drink Types; Table S2: Pre- to Post-tax Levels and Differences in the Percentage of Higher-income Respondents Perceiving Negative Health Consequences of Sweetened Beverage Consumption and Drink Types; Table S3: Difference-in-Differences in the Probability of Answering “I don’t know” to Each of the Perceived Health Impact Questions in Seattle and Comparison Areas; Table S4: Difference-in-Difference in the Probability of Answering “I don’t know” to Each of the Perceived Healthfulness of Sweetened Beverage Type Questions in Seattle and Comparison Areas.
