# Supplementary material for: Impacts of the Seattle Sweetened Beverage Tax on the Perceived Healthfulness of Sweetened Beverages
Source: Nutrients. 2022 Feb 26;14(5):993. doi: 10.3390/nu14050993 (PMC8912807; doi:10.3390/nu14050993)
Supplement: Supplementary file 1 [file nutrients-14-00993-s001.zip › nutrients-1553970-supplementary.pdf]

SUPPLEMENTARY TABLES

Table S1. Pre- to Post-tax Levels and Differences in the Percentage of Lower-income Respondents Perceiving Negative Health Consequences of Sweetened Beverage Consumption and Drink Types[a-c]

Health Impacts & Drink Types	Seattle Pre-Tax (95% CI)	Seattle Post-Tax (95% CI)	Seattle Difference (95% CI)	Seattle P-value of Differences	Comparison Pre-Tax (95% CI)	Comparison Post-Tax (95% CI)	Comparison Difference (95% CI)	Comparison P-value of Differences	Difference of Differences (95% CI)	P-value of Differences
Serious Health	81 (79, 83)	82 (80, 85)	1 (-1, 2)	0.274	91 (87, 94)	79 (76, 83)	-11 (-18, -4)	0.012	12 (5, 19)	0.009
Dental Health	88 (86, 90)	89 (87, 91)	1 (1, 2)	0.001	93 (90, 97)	90 (84, 96)	-4 (-10, 3)	0.184	5 (-1, 12)	0.086
Obesity	85 (83, 87)	90 (88, 93)	5 (3, 7)	0.001	90 (86, 94)	92 (87, 97)	2 (-3, 7)	0.301	3 (-2, 9)	0.192
Diabetes	89 (87, 91)	91 (88, 93)	1 (0, 3)	0.031	94 (88, 99)	86 (84, 88)	-8 (-12, -4)	0.007	9 (5, 13)	0.002
Heart Disease	79 (77, 82)	82 (80, 84)	3 (0, 5)	0.028	84 (80, 88)	79 (74, 85)	-4 (-10, 1)	0.097	7 (2, 12)	0.017
Added Sugar	80 (78, 81)	90 (88, 93)	11 (9, 12)	0	89 (87, 91)	80 (76, 85)	-9 (-13, -5)	0.004	20 (15, 24)	0
Fruit drinks	86 (85, 87)	89 (88, 91)	3 (2, 5)	0.001	86 (81, 92)	88 (83, 93)	2 (-5, 8)	0.494	2 (-5, 8)	0.532
Soda	92 (91, 93)	94 (92, 96)	2 (1, 4)	0.009	93 (90, 96)	93 (86, 100)	-0 (-6, 6)	0.989	2 (-4, 9)	0.385
Sports drinks	79 (77, 81)	81 (79, 84)	3 (2, 3)	0	81 (80, 82)	82 (75, 88)	1 (-7, 8)	0.792	2 (-6, 10)	0.56
Sweetened tea/coffee	85 (82, 88)	88 (84, 92)	3 (2, 4)	0.001	80 (73, 87)	90 (87, 93)	10 (4, 17)	0.012	-7 (-13, -1)	0.034
Energy drinks	91 (90, 91)	89 (88, 91)	-1 (-3, -0)	0.033	91 (86, 96)	92 (88, 95)	1 (-8, 10)	0.838	-2 (-10, 6)	0.529

a. CI = Confidence Interval
b. P < 0.05
c. The estimates in these models were created using population weights combined with propensity score weights. Difference estimates represent changes in the percentage of the population over time, while differences-in-differences estimates represent changes over time in Seattle compared to the changes over time in comparison areas. Units for all estimates are percentage points, rounded to the nearest whole number. Race/ethnicity, education, age, sex, survey mode, and political affiliation were controlled for in each model.

SUPPLEMENTARY TABLES

Table S2. Pre- to Post-tax Levels and Differences in the Percentage of Higher-income Respondents Perceiving Negative Health Consequences of Sweetened Beverage Consumption and Drink Types[a-c]

Health Impacts & Drink Types	**Seattle Pre-Tax (95% CI)**	**Seattle Post-Tax (95% CI)**	**Seattle Difference (95% CI)**	**Seattle P-value of Differences**	**Comparison Pre-Tax (95% CI)**	**Comparison Post-Tax (95% CI)**	**Comparison Difference (95% CI)**	**Comparison P-value of Differences**	**Difference of Differences (95% CI)**	**P-value of Differences**
Serious Health	87 (85, 89)	85 (83, 87)	-2 (-2, -1)	0.001	83 (78, 87)	88 (82, 94)	5 (1, 10)	0.03	-7 (-11, -3)	0.009
Dental Health	89 (87, 90)	91 (90, 92)	2 (2, 2)	0	92 (89, 95)	92 (84, 99)	-0 (-10, 9)	0.899	2 (-7, 12)	0.496
Obesity	87 (86, 88)	91 (90, 92)	4 (3, 4)	0	90 (84, 96)	91 (88, 94)	1 (-8, 9)	0.868	3 (-5, 12)	0.37
Diabetes	88 (87, 88)	92 (92, 93)	5 (4, 6)	0	91 (85, 97)	93 (90, 95)	2 (-5, 8)	0.508	3 (-3, 9)	0.251
Heart Disease	79 (78, 80)	81 (80, 82)	2 (1, 3)	0.003	79 (74, 84)	88 (84, 91)	9 (3, 15)	0.017	-7 (-13, -1)	0.038
Added Sugar	91 (90, 93)	90 (89, 91)	-2 (-2, -1)	0.001	93 (90, 95)	90 (86, 93)	-3 (-8, 2)	0.136	1 (-3, 6)	0.473
Fruit drinks	94 (93, 95)	93 (91, 95)	-1 (-2, -0)	0.028	92 (89, 96)	88 (82, 95)	-4 (-13, 4)	0.237	3 (-5, 12)	0.324
Soda	95 (93, 96)	97 (95, 98)	2 (2, 3)	0	96 (94, 98)	94 (88, 101)	-2 (-8, 4)	0.456	4 (-2, 9)	0.122
Sports drinks	85 (83, 88)	86 (83, 89)	1 (-1, 2)	0.18	83 (76, 89)	85 (80, 90)	2 (-1, 5)	0.131	-1 (-5, 2)	0.367
Sweetened tea/coffee	90 (89, 92)	90 (88, 91)	-1 (-1, -0)	0.026	88 (80, 96)	89 (81, 97)	1 (-2, 3)	0.504	-1 (-3, 1)	0.176
Energy drinks	94 (93, 96)	93 (91, 95)	-1 (-1, -0)	0.01	93 (89, 97)	93 (88, 98)	0 (-1, 2)	0.713	-1 (-2, 0)	0.052

a. CI = Confidence Interval
b. P < 0.05
c. The estimates in these models were created using population weights combined with propensity score weights. Difference estimates represent changes in the percentage of the population over time, while differences-in-differences estimates represent changes over time in Seattle compared to the changes over time in comparison areas. Units for all estimates are percentage points, rounded to the nearest whole number. Race/ethnicity, education, age, sex, survey mode, and political affiliation were controlled for in each model.

SUPPLEMENTARY TABLES

Table S3. Difference-in-Differences in the Probability of Answering "I don't know" to Each of the Perceived Health Impact Questions in Seattle and Comparison Areas[a-e]

Health Impacts	**Lower Income (N = 1364)**	**Higher Income (N = 1729)**
	**DD (95% CI)**	**DD (95% CI)**
Drinking sugary drinks causes serious health problems	1.48 (-1.20, 4.17)	0.77 (-0.70, 2.24)
Drinking sugary drinks significantly raises a person's chances of dental health problems, including cavities and tooth decay	**2.59 (0.30, 4.88)**	1.02 (-1.42, 3.46)
Drinking sugary drinks significantly raises a person's chances of obesity	1.62 (-0.17, 3.42)	0.93 (-1.72, 3.58)
Drinking sugary drinks significantly raises a person's chances of diabetes	0.50 (-0.75, 1.76)	0.28 (-1.58, 2.15)
Drinking sugary drinks significantly raises a person's chances of heart disease	0.31 (-0.62, 1.24)	-0.67 (-1.69, 0.36)
Consuming excessive amounts of sugar from any source can lead to health problems	-0.01 (-0.70, 0.68)	0.22 (-1.12, 1.57)

a. CI = Confidence Interval; DD = Difference-in-difference
b. Bolded values indicate significance at $p < 0.05$; values with an (*) indicate significance of $p = 0.002$, according to the Bonferroni correction
c. Lower income is defined as having an income < 260% FPL; Higher income is defined as having an income ≥ 260% FPL
d. The estimates in these models were created using population weights combined with propensity score weights. Difference-in-differences estimates represent changes over time in Seattle compared to the changes over time in comparison areas. Race/ethnicity, education, age, sex, survey mode, and political affiliation were controlled for in each model.
e. Estimates were created using multinomial logit models. The estimates in the table compare the change over time in the proportion of respondents who answered, 'Don't know' for each of the health impacts questions in Seattle as compared to the comparison area, with those who agreed being the base outcome in the multinomial model.

SUPPLEMENTARY TABLES

Table S4. Difference-in-Differences in the Probability of Answering "I don't know" to Each of the Perceived Healthfulness of Sweetened Beverage Type Questions in Seattle and Comparison Areas[a-e]

Sweetened Beverage Types	**Lower Income (N = 1364)**	**Higher Income (N = 1729)**
	**DD (95% CI)**	**DD (95% CI)**
Drinking fruit-flavored drinks affects a person's chances of developing health problems	0.98 (-0.60, 2.56)	-0.34 (-1.69, 1.01)
Drinking soda affects a person's chances of developing health problems	0.70 (-0.72, 2.12)	-0.96 (-2.19, 0.26)
Drinking sports drinks affects a person's chances of developing health problems	**0.42 (0.00, 0.83)**	**-0.37 (-0.73, -0.00)**
Drinking sweetened teas or coffees affects a person's chances of developing health problems	0.06 (-0.70, 0.81)	**-0.61* (-0.82, -0.40)**
Drinking energy drinks affects a person's chances of developing health problems	0.39 (-0.02, 0.81)	**-0.30 (-0.53, -0.06)**

a. CI = Confidence Interval; DD = Difference-in-difference
b. Bolded values indicate significance at $p < 0.05$; values with an (*) indicate significance of $p = 0.002$, according to the Bonferroni correction
c. Lower income is defined as having an income < 260% FPL; Higher income is defined as having an income ≥ 260% FPL
d. The estimates in these models were created using population weights combined with propensity score weights. Difference-in-differences estimates represent changes over time in Seattle compared to the changes over time in comparison areas. Race/ethnicity, education, age, sex, survey mode, and political affiliation were controlled for in each model.
e. Estimates were created using multinomial logit models. The estimates in the table compare the change over time in the proportion of respondents who answered, 'Don't know' for each of the health impacts questions in Seattle as compared to the comparison area, with those who agreed being the base outcome in the multinomial model.
